# Editorial: Response assessment of radioligand therapies

**DOI:** 10.3389/fonc.2022.904337

**Published:** 2022-07-27

**Authors:** Egesta Lopci, Vikas Prasad

**Affiliations:** ^1^ Nuclear Medicine, IRCCS Humanitas Research Hospital, Rozzano (MI), Italy; ^2^ Department of Nuclear Medicine, University Hospital Ulm, Ulm, Germany

**Keywords:** radioligand therapy, response assessment, PET/CT, neuroendocrine tumors, prostate cancer

Cancer management in the last decades has rapidly evolved and numerous innovative regimens have been implemented to improve patients’ quality of life and survival. Among the extensive list of oncological treatments, radioligand therapy (RLT) has taken over a central role in the management of neuroendocrine tumors and prostate cancer, thanks to the development of beta- or alpha-emitting radiolabeled probes. The outstanding results obtained from randomized multicenter trials (1–4) have granted RLT a solid continuity in cancer treatments and paved the way for future expansion. Just like for immunomodulatory regimens, the universally utilized solid tumor response criteria (i.e. RECIST 1.1 or RECIST 1.0) (5, 6) have proved to be insufficient to face the variety of responses acknowledged with RLT. In fact, these criteria are dependent on the morphological changes measured in two dimensions and lack full assessment of tumor burden in three dimensions. Moreover, response categories defined in RECIST are more rigid and based on the working hypothesis that lesion progression is unidirectional and fixed, which is often contrary to the working principle of treatment with some targeted drugs. Morphological changes induced by targeted drugs, detected by CT or MRI, are often preceded by molecular and histopathological changes. The differences between molecular/histopathological changes and anatomical changes measured by CT become significant for drugs acting *via* direct and indirect interactions with cancer cells, microenvironment and tumor immunity. Phenomena, such as pseudoprogression, may result in the dynamicity of responses measured on CT, and thus need to be properly identified by imagers to help clinicians avoid unnecessary treatment stop or early withdrawal. Unlike immunotherapy, however, the adaptation of response criteria to the new occurrences with RLT has not progressed. Consequently, there is a persisting need to implement molecular and functional criteria into the diagnostic and therapeutic pathway of solid tumors undergoing RLT.

In the current Research Topic we aimed to discuss more thoroughly the problems commonly faced with RLT, including confirmation of disease progression), pseudoprogression (or flare phenomenon), delayed response, optimal timing of response assessment, bone lesion characterization, integration of surrogate biomarkers and PET-based response parameters in CT-based RECIST, as well as resetting the bar in patients candidate to get off RLT, otherwise showing no toxicity and showing clinical and biochemical benefit. Hence, we welcomed Original Research and Review articles that addressed the abovementioned aspects of newly proposed response evaluation criteria.

Pettersson et al. evaluated the role of changes in contrast-enhancement and arterial attenuation in pancreatic neuroendocrine tumors (PNET) liver metastases following peptide receptor radioligand therapy (PRRT). As expected, the maximum arterial tumor attenuation decreased from baseline to follow-up, with no significant changes at early assessment, suggesting that the major biological effects of PRRT require more time to be observed and should be assessed only in the later stages of the therapy.

In the manuscript by Prasad et al. early assessment of response to RLT was performed after 2 cycles of [177Lu]PSMA. Aiming to better detect patients going to benefit from the treatment, the authors enrolled metastasized castration resistant prostate cancer (mCRPC) patients investigated under the German national regulations of compassionate use of a non-approved drug according to AMG §13.2b (German Medicinal Product §13.2b). Therein, response assessment at 8–10 weeks after the 2nd cycle of RLT proved limited role for PSA (prostate specific antigen), whereas interim [68Ga]PSMA PET/CT response resulted predictive of overall survival and progression in patients treated with [177Lu]PSMA.

The advent of new therapeutic options opens the door to clinical validation, and this is the case of irreversible electroporation (IRE) in locally advanced pancreatic cancer (LAPC). This topic was covered by He et al. in their manuscript investigating a total of 312 LAPC patients after IRE treatment with the intent to develop a novel prediction tool or nomogram.

The possibility to enhance the effect of current regimens in neuroendocrine neoplasia (NEN) has been the focus of the article prepared by Exner et al. In particular, the authors dealt with the use of radiosensitization prior to PRRT with mTOR (mammalian target of rapamycin) inhibitors, which is considered a promising strategy to improve the treatment effect. The rationale for the use of these drugs is related to the capability of mTOR inhibitions to arrest the cell growth with a biphasic concentration-response pattern (7). The promising results shown in the paper need further validation in a suitable animal model as well as in therapeutic regimens involving RLT, such as [177Lu]DOTATATE or [177Lu]DOTATOC.

The implementation of MRI is another interesting option in the area of response assessment to RLT. This aspect has been handled by Hu et al. in the last contribution of the Reseach Topic. Therein the percentage change at high-resolution MRI was investigated to assess the association of aggressive tumor response. More specifically, the percentage of tumor invasion (PTI) obtained on MRI could serve as an imaging biomarker of tumor aggressiveness and predict therapeutic benefit. Although the authors focused their research on T3 rectal cancer undergoing neoadjuvant chemoradiation, the concept is easily translated to other therapeutic regimens. Giving for granted the fact that all new imaging biomarkers should undergo prospective validation, possibly within randomized clinical trials.

The need for standardization of response evaluation criteria for radioligand therapy has also been taken up seriously by some of the international guideline makers e.g. European Neuroendocrine Tumor Society (ENETS). The final aim is to first understand the basic mechanism of action of radioligand therapies at the cellular and subcellular level. Functional imaging allows decoding these changes, however, they need to be standardized and optimized. More importantly, reliable clinical endpoints and biochemical markers need also to be integrated in the response evaluation criteria guiding therapy management.

## Author contributions

All authors listed have made a substantial, direct, and intellectual contribution to the work and approved it for publication.

## Acknowledgments

The authors would like to thank Prof. Richard Baum and Dr. Lisa Bodei for the support in the preparation of this Research Topic.

## Conflict of interest

The authors declare that the research was conducted in the absence of any commercial or financial relationships that could be construed as a potential conflict of interest.

## Publisher’s note

All claims expressed in this article are solely those of the authors and do not necessarily represent those of their affiliated organizations, or those of the publisher, the editors and the reviewers. Any product that may be evaluated in this article, or claim that may be made by its manufacturer, is not guaranteed or endorsed by the publisher.
